# Influence of Soil Type, Cultivar and *Verticillium dahliae* on the Structure of the Root and Rhizosphere Soil Fungal Microbiome of Strawberry

**DOI:** 10.1371/journal.pone.0111455

**Published:** 2014-10-27

**Authors:** Srivathsa Nallanchakravarthula, Shahid Mahmood, Sadhna Alström, Roger D. Finlay

**Affiliations:** Uppsala BioCenter, Department of Forest Mycology and Plant Pathology, Swedish University of Agricultural Sciences, Uppsala, Sweden; Kansas State University, United States of America

## Abstract

Sustainable management of crop productivity and health necessitates improved understanding of the ways in which rhizosphere microbial populations interact with each other, with plant roots and their abiotic environment. In this study we examined the effects of different soils and cultivars, and the presence of a soil-borne fungal pathogen, *Verticillium dahliae*, on the fungal microbiome of the rhizosphere soil and roots of strawberry plants, using high-throughput pyrosequencing. Fungal communities of the roots of two cultivars, Honeoye and Florence, were statistically distinct from those in the rhizosphere soil of the same plants, with little overlap. Roots of plants growing in two contrasting field soils had high relative abundance of *Leptodontidium* sp. C2 BESC 319 g whereas rhizosphere soil was characterised by high relative abundance of *Trichosporon dulcitum or Cryptococcus terreus*, depending upon the soil type. Differences between different cultivars were not as clear. Inoculation with the pathogen *V. dahliae* had a significant influence on community structure, generally decreasing the number of rhizosphere soil- and root-inhabiting fungi. *Leptodontidium* sp. C2 BESC 319 g was the dominant fungus responding positively to inoculation with *V. dahliae.* The results suggest that 1) plant roots select microorganisms from the wider rhizosphere pool, 2) that both rhizosphere soil and root inhabiting fungal communities are influenced by *V. dahliae* and 3) that soil type has a stronger influence on both of these communities than cultivar.

## Introduction

Plants roots influence the structure and diversity of soil microbial communities through their energy-rich exudates [1], [2], creating diverse rhizosphere communities in close proximity to the roots [3]. The roots are colonized by a subset of these microorganisms, creating both a rhizoplane community on the surface and an endophytic compartment. Discrimination between pathogens and beneficial mutualists or commensals is controlled by an immune system [4], but the mechanisms underlying the selection of different core microbiomes [5] by different plant species are still poorly understood. Recent studies of bacteria associated with the roots or rhizosphere of *Arabidopsis thaliana* grown in geochemically distinct soils [6] have shown that they are both strongly influenced by soil type but that the root endophytic compartments from two soils contain overlapping, low complexity communities that are enriched in Actinobacteria and specific families belonging to the Proteobacteria. Differential relative abundance of *Actinocorallia* sp. has also been shown in different ecotypes of *A. thaliana* [7].

Plant host genetics plays a significant role in the selection of microbial communities associated with roots [8]–[10]. This selection has important implications for plant health and growth since rhizosphere microorganisms are known to have a variety of positive or negative effects. These include plant growth stimulation, antagonism against pathogens and effects on carbon sequestration and phytoremediation. Rhizosphere and root endophyte microorganisms are increasingly being used on a commercial scale for plant protection as biocontrol agents and biofertilizers [11]–[13]. Management of such microorganisms requires a thorough understanding of their interaction with other microorganisms sharing the same ecological niche. Some attempts have been made to study the effect of biocontrol agents on microbial communities [14]–[17], however studies focusing on the influence of soil-borne fungal plant pathogens on microbial community structures are less frequent [18]–[21]. Most of the inoculation studies with pathogens or biocontrol agents have been more focused on bacterial communities than on fungal communities.

The rhizosphere is a dynamic region for many biological processes and interactions [1], [11], [12], [22]–[25] and empirically-based management strategies for sustainable cultivation need to be based on a more rigorous understanding of the functional basis of the mechanisms underlying the various biotic interactions taking place [26].

Microbial community structure in the rhizosphere and the root endophytic compartment is shaped by soil characteristics as well as by plant genotype [6], [7], [20], [27]–[31]. Soils in organically managed strawberry fields have been shown to have greater functional activity and higher functional gene diversity than soils in conventionally managed fields [32]. The strawberry rhizosphere has been shown to harbour distinct microbial communities containing *Streptomyces*, *Rhizobium* and *Nocardia* [28], [31] and in the presence of *Verticillium dahliae,* a soil-borne fungal pathogen, it is dominated by *Pseudomonas* spp. populations [27]. Studies have also shown that strawberry cultivars respond differently to the inoculation of arbuscular mycorrhizal fungi (AMF) [33], [34]. Sporulation of *Phytophthora fragariae* associated with strawberry plants colonized by AMF has also been shown to be reduced in comparison to that in non-AMF colonized plants [35].

Strawberry (*Fragaria*×*ananassa*) is grown for its berries in many countries and different cultivars have been developed for commercial production. Rhizosphere bacterial biomass and activity, fruit quality and yield have all been shown to vary under different management systems. Soil microorganisms have been shown to be indirectly involved in determining the fruit quality [32], [36]. *Verticillium dahliae* is one of several plant pathogens that affect plant growth and yield in different plant species, including strawberry [37]–[41]. The pathogen forms conidia and microsclerotia that germinate in the presence of the root exudates, whereupon germ tubes enter the plant roots. The pathogen can persist as microsclerotia on dead plant tissues, or in the soil for more than 10 years [39]–[41]. In strawberry as few as two microsclerotia per gram of soil can cause 100% wilt [42] and this pathogen is difficult to combat with current management strategies. In a field trial, biocontrol using *Trichoderma harzianum* and *Trichoderma viride* against *V. dahliae* reduced wilting in strawberry by 60%. An indirect effect against *V. dahliae* through induced systemic resistance by *Paenibacillus alvei* K165 has also been observed in *Arabidopsis* sp. [43], [44].

Studies have shown that the presence of fungal (*Phytophthora cinnamomi*) and bacterial pathogens, (*Liberibacter asiaticus* and *Erwinia carotovora* (*Pectobacterium carotovorum*)) increases the diversity of Bacteroidetes and Alphaproteobacteria in particular [18], [20], [21]. Such changes also facilitate the selection, by the plant, of taxa including those with antimicrobial properties [27], [45].

Plant resistance is one of the effective strategies in plant protection and attempts have been made to study effects of resistant and susceptible cultivars on microbial communities [46]–[48]. The results of these studies demonstrate that plant genotype can have a significant impact on soil microbial community structure, and differences in the rhizosphere microbial community have been suggested to contribute to the differences in resistance to disease. Whether this impact is independent of the soil type is not evident from these studies.

Many earlier studies involving soil microbial community analyses have been conducted using either cultivation-dependent approaches or molecular methods such as T-RFLP, DGGE, cloning and Sanger sequencing which are now proven to underestimate the total microbial diversity [49], [50]. More recent studies using high throughput techniques have revealed taxa that were not detected with previous methods [51].

In this study we investigated the impact of plant genotype on the fungal community structure in different soil types in the presence and absence of the soil-borne fungal pathogen, *V. dahliae*. We used 454 pyrosequencing to analyze the rhizosphere soil- and root fungal communities in different strawberry cultivars, grown in differently managed soils. We hypothesized that the structure of microbial communities in the rhizosphere soil and roots is determined by both the cultivar and soil type, and may also be re-structured in the presence of the pathogen.

## Materials and Methods

### Experimental soils and plant material

Four cultivars of strawberry, Florence, Honeoye, Senga Sengana, and Zephyr, and two of the three different soils used in this study were the same as those described in a previous study by Santos-González et al. [30]. The cultivars were selected on the basis of their breeding year, yield level and disease tolerance. Honeoye and Florence were bred after 1978, Zephyr and Senga Sengana before 1966. Honeoye is considered to be a high yielding cultivar and Florence and Senga Sengana are considered to be tolerant to *V. dahliae* [52]–[54]. In our figures the conventionally managed soil from Kristianstad is referred to as “conventional” and the organically managed soil from Hörby as “organic” [30]. The field in Hörby was managed organically with appropriate crop rotations since 1983 and pre-cropped with potatoes prior to sampling in 2009. The experimental field in Kristianstad is conventionally managed and was planted with strawberry in 2008. The two agricultural fields are located 38 km apart in Southern Sweden. These sites are on private land and permission to collect soil was obtained from the owners Sune Abrahamsson (Kristianstad) and Ingvar Åkesson (Hörby). The soil collection did not involve endangered or protected species. The Kristianstad site at (56° 06′ N, 14° 01′ E) and the Hörby site at (55° 50′ N, 13° 35′ E) have different soil properties. The physico-chemical traits of the two field soils are described by Santos-González et al. [30]. The soils had similar textural properties (8.5–11% clay, 29–35% silt, 51–58% sand) and bulk density (1.05–1.07 g cm^−3^). However the pH, C, N and P levels of the Hörby soil were significantly (*P*<0.05) higher than those of the Kristianstad soil (6.36 vs. 5.97; 2.1% vs. 1.61%; 0.17% vs. 0.14%; 181 mg kg^−1^ vs. 113 mg kg^−1^). The third “soil” was a peat-based growth substrate (Hasselfors Garden Special, Hasselfors Garden AB, Örebro, Sweden) containing 60% light peat, 25% black peat, 15% sand (0.5–4 mm) and 0.050 kg m^−3^ multisport/FTE36, 2.0 kg m^−3^ dolomite and 4.5 kg m^−3^ limestone, amended with 1.3 kg m^−3^ fertilizer (N14:P7:K15, pH-6.0), and is used for commercial greenhouse cultivation in Sweden.

### Pathogen inoculum

The pathogen isolate used was *V. dahliae* 12086, originating from strawberry roots and available from the Food and Environment Agency, York, UK (see http://www.q-bank.eu/). It was purified and grown in potato dextrose broth (PDB per L, 12 g Accumedia, Michigan, USA) as stationary cultures for 20 days at 24°C. The mycelial mat thus obtained was macerated carefully in diluted potato dextrose broth (1:10 water v/v). A preparatory study was conducted with the suspension thus obtained to confirm the pathogenicity of the test pathogen. On the basis of the results from the preparatory study (data not shown), 30 ml mycelial suspension was inoculated in *V. dahliae*-treatments. The plant roots were injured by inserting a 0.9 cm diameter cork borer into the root volume in three locations to stimulate the pathogen infection. No dead-cell, or chitin controls were used but the control plants without the pathogen were treated in a similar manner but with suspension medium not containing fungal mycelium.

### Experimental design

An outdoor experiment was conducted by growing the four cultivars in the three soils as specified in a previous study [30]. In brief, the soils were prepared before planting with one plantlet per pot (13×13 cm×25 cm deep), placed outdoors and irrigated as needed. The plantlets were taken from storage under refrigeration and were about 5 cm tall with three to four leaves and roots about 15 cm long.

The cumulative fresh berry weight per plant was recorded during the experimental period of 14 weeks. The rhizosphere soil was collected from three replicates 12 weeks after planting and 14 weeks after planting. To sample the rhizosphere soil, the plants were carefully removed from the pots and roots with tightly adhering soil were suspended in phosphate-buffered saline solution (PBS, 0.14 M NaCl, 0.0027 M KCl, 0.010 M phosphate buffer, pH 7.4, Medicago, Sweden) for 30 minutes. The rhizosphere soil thus collected was centrifuged at 8000 RPM for 10 minutes at 4°C (Biofuge PrimoR, Heraeus Sorvall), the roots were once again washed carefully under aerated tap water. The soil pellet and the roots were stored at −20°C for further analyses.

### Analysis of fungal communities

#### DNA extraction and selection of samples for pyrosequencing

DNA extraction methods differ in their efficiency of extraction from different taxa and for the rhizosphere samples, we used two different nucleic acid extraction methods and the resultant DNA extracts were checked individually for quality and reproducibility on a DGGE (denaturing gradient gel electrophoresis) gel following PCR amplifications. The first method was based on ‘CTAB’ (hexadecyltrimethylammonium bromide) [55] that involved extraction of nucleic acids from 0.5 g of soil. The soil samples were weighed in 2 ml ‘Lysing matrix B’ tubes (Fisher Scientific, USA) containing 0.5 ml CTAB buffer (120 mM and pH 8.0) and 0.5 ml phenol: chloroform: isoamylalcohol (25:24:1 V/V). The cells were lysed at 5000 RPM for 30 seconds in a bead beater (Precellys 24; Bertin Technologies, France). Proteins and solvent residues were removed using chloroform and isoamyl alcohol (24:1) followed by further precipitation using polyethylene glycol (30%). The precipitated DNA was washed with ice-cold ethanol (70%), air dried and re-suspended in 30 µl of MilliQ water and stored at −20°C for further analysis.

The second nucleic acid extraction method was based on MOBIO ‘RNA power soil’ total RNA isolation and DNA elution accessory kits (MOBIO laboratories, California, USA) according to manufacturer’s recommendations. The concentration and quality of extracted DNA were assessed using NanoDrop (ND-1000 Nanodrop technologies, USA). The banding profiles of these two methods differed so the DNA from these two extraction methods was pooled and used as a mixed template for generating PCR amplicons for pyrosequencing. Prior to pyrosequencing, the control samples were subjected to DGGE-based analysis following PCR amplification. The DGGE banding profiles showed higher numbers of bands at the first sampling, 12 weeks after planting, than at 14 weeks (data not shown). Pyrosequencing was therefore carried out on samples from 12 weeks.

For the analysis of root-associated fungi we chose to analyze two cultivars Honeoye, susceptible to *V. dahliae*, and Florence, tolerant to *V. dahliae*, growing in each of the two field soils. Nucleic acids from roots were extracted using DNeasy plant mini kit (Qiagen, Germany) according to the manufacturer’s recommendations. Roots were carefully washed to remove the soil particles attached to the roots and soil removal was microscopically confirmed. They were gently washed three times with 0.1% Triton x100 for two minutes, thereafter washed with sterile milliQ water. All collected roots were subsequently freeze-dried for 48 h (CoolSafe, ScanLaf A/S, Denmark), homogenised by milling in 2 ml tubes containing 2.38 mm diameter stainless steel beads (MOBIO laboratories, California, USA) in a bead-beater for 30 s twice. 50 mg of the homogenised root material was used for nucleic acid extraction. The extracted DNA was quantified and used as a template for generating PCR amplicons for pyrosequencing.

### Pyrosequencing

Pyrosequencing analysis of the fungal communities was carried out as described by Ihrmark et al. [56] using primers fITS7 (5′GTGARTCATCGAATCTTG-3′) and ITS4 (5′TCCTCCGCTTATTGATATGC-3′) [57]. In brief, each 50 µl reaction mixture consisted of 200 µM of dNTPs, 2.75 mM MgCl_2_, primers at 500 nM and 300 nM, 0.025 U polymerase (DreamTaq Green, Thermo Scientific, Waltham, MA) in buffer. The thermocycling conditions were: 5 min at 94°C; 35 cycles (30 s at 94°C; 30 s at 57°C; 30 s at 72°C); 7 min at 72°C. Three technical replicates were run for each sample. To ascertain PCR amplification efficiency and to rule out any possible contamination, all reactions were run with positive and negative controls. The PCR amplification products were analyzed on 1% (w/v) agarose gels pre-stained with Nancy red-520 (Sigma Aldrich, USA). The triplicate PCR products of each sample were pooled, purified using Agencourt AMPure, PCR purification kit (Beckman Coulter, Massachusetts, USA) and quantified using a Qubit Fluorometer (Invitrogen, USA). Equimolar concentrations of samples were pooled and the resultant mixture was freeze-dried (CoolSafe, ScanLaf A/S, Denmark) overnight. Pyrosequencing was carried out on 2×1/8th of a GS FLX Titanium Pico Titer Plate (LGC Genomics, Germany and MACROGEN, South Korea) according to the manufacturer's instructions (Roche, Branford, CT).

### Bioinformatic analysis

The SCATA pipeline (Sequence Clustering and Analysis of Tagged Amplicons, http://www.scata.mykopat.slu.se) was used to analyze the sequence data from 72 rhizosphere samples and 24 root samples. The quality control and data filtering involved removal of singletons, low quality sequences, tags and primer missing sequences, followed by removal of sequences shorter than 200 base pairs (after primer and tag trimming). We used default parameters of the SCATA pipeline except that the proportion of primer match was set at 0.9. Sequences were assembled into clusters by single-linkage clustering with a 98.5% sequence similarity threshold. The most common genotype/sequence of the cluster was used to represent the operational taxonomic units (OTUs). All the representative sequences of the clusters were identified using the NCBI (National Center for Biotechnology Information) database and the nucleotide blast ‘BLASTn’ program. The most common sequence from each cluster was analyzed manually for blasting. With the exception of rarefaction analysis, only sequences with ≥95% identity were chosen for these analyses.

The pyrosequencing data have been deposited at ENA (European Nucleotide Archive) under accession number PRJEB6260.

### Statistical analyses

Statistical analyses were conducted using a significance level of *p*≥0.05. The Chao-1 index (PAST (PAleontological STatistics), version 2.17b) was calculated to obtain the number of OTUs present in the four cultivars grown in three different soils with and without *V. dahliae*. The bar diagrams were generated using Microsoft Excel and the significance of the OTU data was estimated using ANOVA with MINITAB. The relative abundance of OTUs was used for Non-metric multi-dimensional scaling (NMDS) ordination performed with ‘PAST’ using Bray-Curtis dissimilarity indices. Numbers of OTUs were calculated for selected phyla and plotted in Microsoft Excel and their significance was calculated by using ANOVA. SIMPER (similarity percentage) analysis (PAST ver 2.17b) was performed using relative abundance data in order to identify the OTUs that were contributing to the differences among treatments. The rarefaction was estimated using Analytic Rarefaction (Ver 1.3, UGA Stratigraphy Lab, USA).

## Results and Discussion

Bioinformatic analysis of root and rhizosphere material associated with the cultivars Honeoye and Florence revealed a total of 577 unique OTUs following quality control filtering and removal of singletons. In total 360 OTUs were associated with rhizosphere soil and 307 OTUs were associated with roots, with an overlap of 90 OTUs between the two compartments. Analysis of the rhizosphere soil associated with roots of all four cultivars revealed 128 OTUs common to both the field soils but only 16 fungal OTUs that were common to all three soils. In conventionally managed soil 63 OTUs were common to all four cultivars, with 28 common OTUs in organically managed soil and 18 common OTUs in peat-based growth substrate.

**Figure 1:**
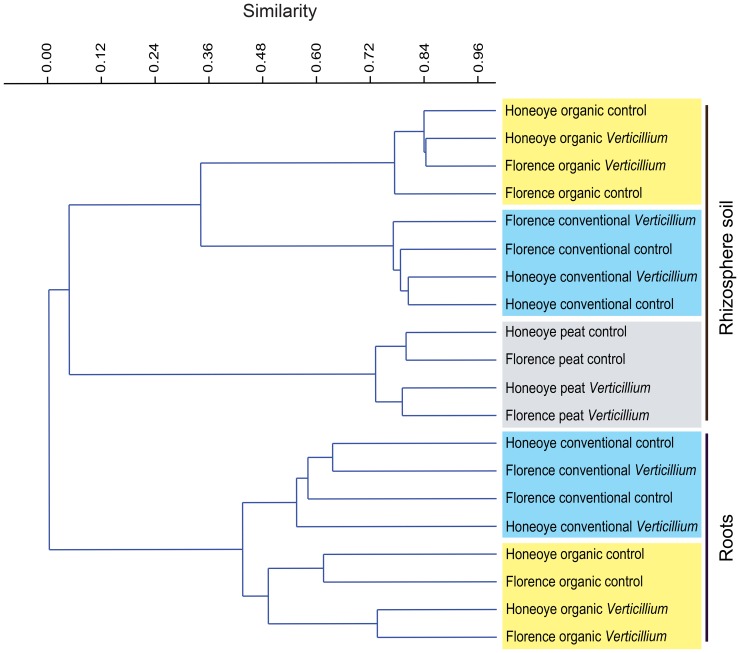
Cluster analysis of the 50 most abundant fungal operational taxonomic units (OTUs) in the rhizosphere and roots of two strawberry cultivars, Honeoye and Florence, grown in conventionally or organically managed soils or a peat based growth substrate, with and without *Verticillium dahliae*. These OTUs constituted 90% of the total reads in roots and 70% of the reads in rhizosphere soil. The clustering is based on paired group linkage using the Bray-Curtis similarity measure. Only OTUs with ≥95% identity are included.

Cluster analysis of the community structure of the 50 most abundant fungal taxa was performed. These taxa constituted 90% of the reads in roots and 70% of the reads in rhizosphere soil. The analysis (Figure 1) revealed a clear separation between fungal communities from the rhizosphere soil and those from the roots. Within each of these compartments there was a clear separation according to soil type (ANOSIM *p = *0.0001). The root associated community structure of the organically managed soil showed a statistically significant (ANOSIM *p* = 0.015) separation between *Verticillium* inoculated and uninoculated treatments. Rhizosphere soil communities in a peat-based substrate formed a distinct cluster with a clear separation between *V. dahliae* inoculated and equivalent, non-inoculated treatments. In a similar analysis of rhizosphere soil communities associated with four different strawberry cultivars, Honeoye, Florence, Senga-Sengana and Zephyr (Figure S1) clear effects of soil type were visible. Differences due to cultivar were less evident. These results are consistent with the finding of Santos-González *et al* [30] that soil type, but not cultivar, shapes the assemblages of arbuscular mycorrhizal fungi associated with strawberry roots in these soils.

Additional NMDS analysis of the fungal community structure in the rhizosphere soil of all four cultivars (Figure S2) showed a statistically significant effect of *Verticillium* inoculation in conventionally managed soil (ANOSIM *p* = 0.005) and peat based substrate (ANOSIM *p* = 0.0004). No such clear separation was evident in organically managed soil.

**Figure 2:**
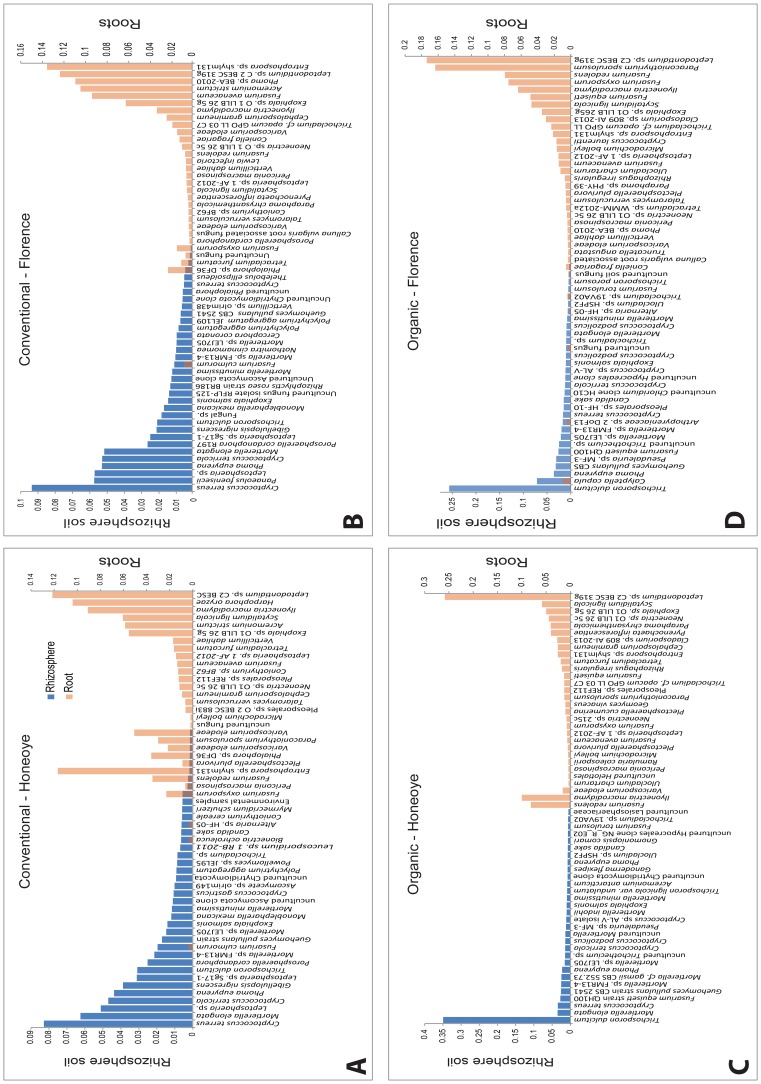
Ranked abundance of the 30 most abundant fungal operational taxonomic units (OTUs) present in the rhizosphere and roots of two strawberry cultivars, Honeoye and Florence, grown in conventionally (A and B) and organically (C and D) managed field soils. Only OTUs with ≥95% identity are included.

Rank abundance plots of the 30 most abundant fungal taxa in each of the rhizosphere and root compartments in the absence of *Verticillium* (Figure 2) revealed that the structure of the fungal microbiome in these two compartments is markedly different, with little overlap. The dominant OTUs from the roots were found at low frequencies in the rhizosphere, while OTUs that were dominant in the rhizosphere were absent or occurred at very low frequencies in the roots, in both field soils (Figure 2a–d). These results are in agreement with other recent studies of bacterial microbiomes of *Arabidopsis thaliana* and the fungal microbiome of *Pisum sativum* [6], [7], [58] that also show distinct communities in the rhizosphere and root endophyte compartments. Within each soil this pattern was the same for both Honeoye and Florence, however there were large differences between the rank abundance distributions of taxa between the two soils. The same lack of overlap between root and rhizosphere soil fungal communities was found in the presence of *Verticillium* (data not shown).

The top seven most abundant taxa in conventionally managed rhizosphere soil, irrespective of strawberry cultivar were *Cryptococcus terreus*, *Mortierella elongata*, *Cryptococcus terricola*, *Panaeolus foenisecii, Phoma eupyrena*, *Gibellulopsis nigrescens* and an unidentified *Leptosphaeria* species, together accounting for 33% and 37% of the total reads for the Honeoye and Florence treatments respectively. In the organically managed rhizosphere soil the top six fungal taxa accounted for 50% of the reads but *Trichosporon dulcitum* was the dominant taxon, accounting for 35% and 30% of the total reads in the Honeoye and Florence treatments respectively. *Trichosporon dulcitum* was also detected in the conventionally managed soil, but at much lower frequencies (3% and 2%). *Trichosporon dulcitum* is a soil-borne basidiomycetous yeast. Utilization of benzene compounds [59] and biodegradation of phenol [60] have been shown in *T. dulcitum*, but the ability of this yeast to interact with plants and fungal pathogens is still unclear. Other members of the Trichosporonales have been found in the rhizosphere and ectomycorrhizosphere in soil from a *Nothofagus pumilio* forest [61]. In the latter study *T. dulcitum* was found in the bulk soil but not in the rhizosphere and no other information appears to be available concerning the possible associations of this species with plant roots.

The most common fungal taxa found in roots included *Entrophospora* sp shylm 131, *Leptodontidium* sp. C2 BESC 319 g, *Ilyonectria macrodidyma*, *Fusarium* spp., *Phoma* sp BEA-2010, *Scytalidium lignicola*, *Acremonium strictum*, *Exophiala* sp. O 1 LILB 26 5 g, and *Paraconiothyrium sporulosum.* The composition of fungal taxa in roots of plants growing in the organically managed soil was characterised by high abundance of *Leptodontidium*, whereas both *Leptodontidium* and *Entrophospora* were dominant in roots of plants growing in the conventionally managed soil. Differences in the abundance of fungal taxa in relation to soil types and strawberry cultivar are discussed below in a separate SIMPER analysis.

**Figure 3:**
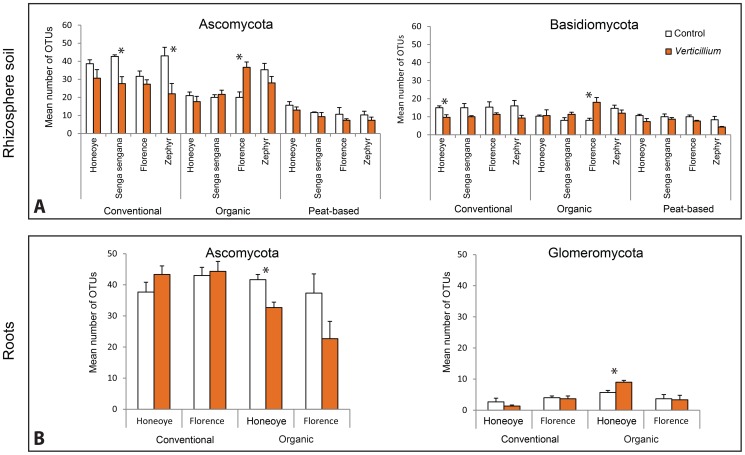
Distribution of fungal operational taxonomic units (OTUs) between different fungal phyla in the rhizosphere (A) and roots (B) of different strawberry cultivars grown in conventionally or organically managed soils or a peat-based growth substrate with and without *Verticillium dahliae*. Only OTUs with ≥95% identity are included. Asterisks indicate statistical significance between control and *Verticillium dahliae* treatments within each soil (* = p≤0.05, ANOVA). Vertical bars represent mean values and error bars show ±1 SE (n = 3).

The total numbers of fungal OTUs in the absence of *Verticillium* inoculation, were generally highest in the rhizosphere of plants grown in conventionally managed soil, intermediate in organically managed soil and lowest in peat-based growth substrate, indicating a strong effect of soil type on fungal diversity. The lowest fungal diversity in the peat based growth substrate is most likely due to its simple composition [62]. In the presence of *Verticillium* no clear difference between the field soils was evident but numbers of OTUs were lower in the peat-based growth substrate (ANOVA *p*≤0.05) (Figure 3a).

In conventionally managed soil, *Verticillium* inoculation resulted in a consistent reduction (20–45%) in the number of fungal OTUs in the rhizosphere that was statistically significant (*p*≤0.05) in Senga Sengana and Zephyr (Figure 3a). In contrast, in the organically managed soil, inoculation with *Verticillium* resulted in significant (*p*≤0.05) increases (114% and 125%) in the total numbers of ascomycete and basidiomycete OTUs respectively in the rhizosphere of Florence (Figure 3a). The reasons for this increase are not clear but may reflect differences in the quality and quantity of root exudates of Florence, which is considered to be a *Verticillium* tolerant cultivar [63]. Basidiomycota were less abundant in the rhizosphere but also showed a consistent reduction in response to *Verticillium* in plants grown in conventionally managed soil. This was statistically significant only in Honeoye, but evident as a trend in the other cultivars.

Ascomycota dominated, both in roots and in rhizosphere (Figure 3a & b). *Verticillium* inoculation had no statistically significant effects on the number of Ascomycotan OTUs in roots of Honeoye and Florence grown in conventional soil but these were significantly reduced by 21% in Honeoye in response to *Verticillium* inoculation in organically managed soil. The mean number of Ascomycotan taxa in the roots of Florence was 38% lower in the presence of *Verticillium* but this reduction lacked statistical support.

Numbers of Basidiomycotan OTUs in roots were negligible, but 17 Glomeromycotan OTUs were found, including *Entrophospora* sp. shylm 131, *Rhizophagus intraradices* and *Rhizophagus clarus*. Numbers of Glomeromycotan OTUs in Honeoye roots in organically managed soil were significantly increased by 50% (Figure 3b) in response to *Verticillium* inoculation. Xu et al [58] have also shown that the relative abundance of different fungal phyla varies in root and rhizosphere samples of *Pisum sativum* depending upon whether the plant is healthy or infected with the pathogen *Aphanomyces euteiches.*

Rarefraction analysis of the numbers of OTUs associated with different numbers of sequences from root and rhizosphere samples are shown in Figure S3. The root treatment samples approached saturation, with asymptotes between 80 and 120 OTUs, although fewer root treatments were analysed in this study since only the roots of Honeoye and Florence plants were examined. The species accumulation curves for rhizosphere soil samples included three growth substrates and four cultivars and were not saturated, suggesting that not all taxa were detected. However there was a high degree of consistency between the most abundant taxa in the rhizosphere for both soils (Figure 2), suggesting that the dominant taxa in these communities were adequately described.

SIMPER (Similarity Percentage) analysis of OTUs from rhizosphere and root samples was performed to identify the species primarily contributing to the difference in community structure between the two compartments and the results are summarized in Table S1.

In Florence the effect of *Verticillium* inoculation on fungal community structure was stronger in roots (71% in organically managed soil, 60% in conventionally managed soil) than in the rhizosphere, (46% in organically managed soil, 52% in conventionally managed soil). In Honeoye the contribution to *Verticillium* induced differences was lower but the values for roots (53% in organic soil and 54% in conventionally managed soil) were still higher than in the rhizosphere (41% in organic soil and 48% in conventionally managed soil).

The numbers of reads matching *Verticillium* genera were 564 in roots compared to only 35 in the rhizosphere soil suggesting that *V. dahliae* was more abundant in the roots than the rhizosphere. This is not surprising since *V. dahliae* is a root pathogen but the low number of reads in rhizosphere soil may reflect primer bias against *V. dahliae* or low template concentrations at the time of sampling. Van der Mark et al [64] reported detection of significantly more pathogen DNA of *Aphanomyces euteiches* and *Phytophthora medicaginis* in a susceptible variety of alfalfa than in the resistant variety [64]. In our study, in the conventionally managed soil, significantly higher numbers of reads (345) matching *Verticillium* were detected in *Verticillium* inoculated treatments of Honeoye, the susceptible cultivar, than in Florence, a tolerant cultivar (17 reads). In the organically managed soil the numbers of reads were lower than 17 in both cultivars, suggesting that this soil was possibly suppressive in character, while the conventionally managed soil was more conducive to *Verticillium*.

**Figure 4:**
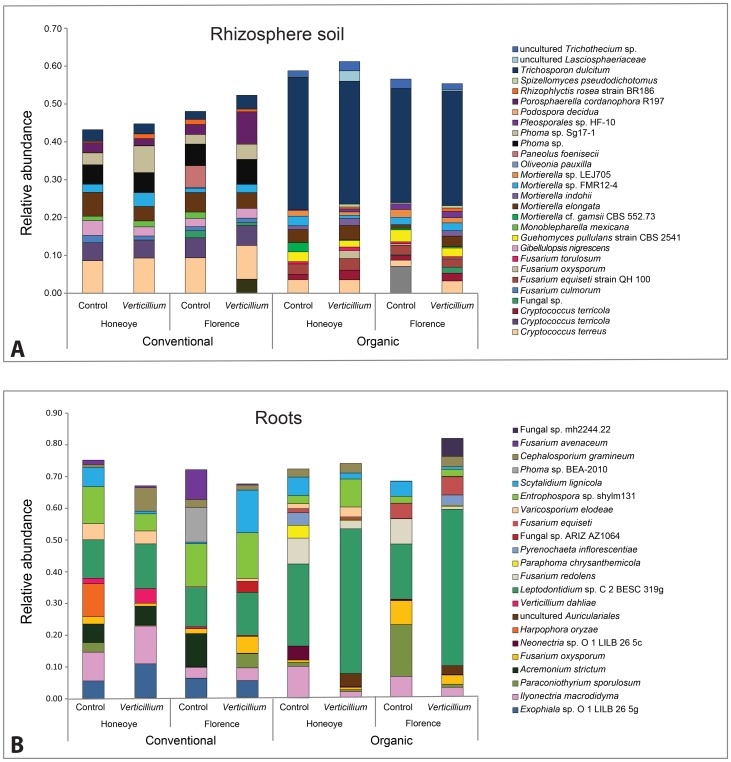
The top ten fungal operational taxonomic units (OTUs) (according to Similarity Percentage analysis) (SIMPER) contributing to the observed differences between control and *Verticillium dahliae* treatments in Honeoye and Florence grown in conventionally and organically managed soils. A) Rhizosphere soil (29–39%), B) Roots (61–74%).

The top ten OTUs contributing to the differences between control and *Verticillium* treatments across all treatments, identified using SIMPER analysis, are shown in Figures 4a–b. The dominant OTUs in roots occur at low frequencies in the rhizosphere soil while OTUs that dominate in the rhizosphere soil are either absent or occur at low frequencies in the roots. This discrepancy between root and rhizosphere soil communities is in agreement with recent observations in other plants [6], [7], [58]. *Cryptococcus* spp., *Gibellulopsis* sp., *Mortierella* spp. and *Phoma* sp. were abundant in rhizosphere in the conventionally managed soil while *Trichosporon dulcitum* and *Fusarium equiseti* were abundant in rhizosphere of organically managed soil. In contrast, *Leptodontidium* sp. dominated the roots in organically managed soil, while *Entrophospora* sp. dominated the roots of plants in conventionally managed soil (Figure 4a, b).

*Verticillium dahliae* was among the top ten species contributing to differences in fungal community structure between inoculated and uninoculated roots of Honeoye plants growing in conventionally managed soil and had a higher relative abundance in inoculated treatments than in uninoculated treatments (1.7%, 4.6%). The relative abundance of *Verticillium dahliae* was much lower in roots of Florence plants (0.56%, 0.27%). This result is consistent with the greater tolerance of Florence to *Verticillium* than Honeoye. The fungus was not present amongst the top ten taxa in organically managed soil, irrespective of strawberry cultivar or inoculation treatment.

*Leptodontidium* sp C2 BESC319g was one of the main contributors to differences in community structure in response to inoculation with *Verticillium*. In conventionally managed soil its relative abundance varied between 12 and 14% and was not affected by *Verticillium* inoculation. However in organically managed soil its relative abundance was much higher, increasing from 28.5% to 45.6% in Honeoye and from 17.3% to 49% in Florence, in the presence of *Verticillium* inoculation. This suggests that *Leptodontidium* sp C2 BESC319g may be one of the biological factors associated with the suppressive character of the organically managed soil. *Leptodontidium orchidicola* has been shown to decrease the negative effect of *V. dahliae* in tomato, but only at low levels of pathogen inoculation [65].

Root endophytes are known to produce secondary metabolites that may have anti-microbial effects on plant pathogens and biological control of *Verticillium dahliae* by root endophytes has been successfully demonstrated [66]–[70]. In the present study the absence of *V. dahliae* associated with increased abundance of *Leptodontidium* in roots of plants grown in organically managed soil, suggests that this fungus may be of functional significance in protection against vascular pathogens. Further research on root endophytic communities should provide new biological control strategies for improving plant health [25].

OTUs of other broadly pathogenic taxa were also detected in our study. Examples of these include species belonging to the genera *Scytadilium, Fusarium, Pyrenochaeta, Phoma and Cephalosporium* but their pathogenicity to strawberry remains largely unknown. The relative abundance of these taxa was affected by both cultivar and soil type in the presence of *Verticillium* (Figure 4 a–b).

The effect of soil type, cultivar and pathogen inoculation on OTUs was also reflected in the plant performance measured as berry yield in this study (Figure S4). The strawberry yield differed significantly between the four cultivars in all three soils (conventional *p* = 0.0014, organic *p* = 0.0087 and peat *p* = 0.0022) in the absence of *V. dahliae*. Honeoye and Zephyr were selected because of their high yielding potential. In the present study these two cultivars produced the highest yields in the conventionally managed soil and peat based growth substrates but their mean yields decreased in response to inoculation with *V. dahliae*. The response of Florence to inoculation with the pathogen varied according to the soil type demonstrating a significant positive effect in conventionally managed soil and a significantly negative response in organically managed soil. Senga Sengana had the poorest yields in all three soils irrespective of the presence or absence of *V. dahliae* (Figure S4).

### Concluding remarks

The results of this study show a marked difference between the fungal community structure in rhizosphere soil and that of fungi growing on or within the root tissues of strawberry plants. This suggests that plant roots select microorganisms from the wider rhizosphere soil pool, but further experiments with sampling at multiple time points are necessary to shed further light on this phenomenon. Strong differences in community structure were also observed between different soils, but differences between fungal communities colonizing roots of different cultivars were less clear. Inoculation with the pathogen *V. dahliae* had a significant influence on community structure and decreased the number of rhizosphere soil- and root-inhabiting fungi in several treatments. Lower numbers of *Verticillium* reads were detected in the roots of Florence, a tolerant cultivar, than in roots of Honeoye. Significant decreases in the number of ascomycete fungal OTUs occurred in roots of both Honeoye and Florence cultivars in response to *V. dahliae* in organically managed soil. The rhizosphere soil of these plants was characterised by high relative abundance of *Trichosporon dulcitum*, while the roots were dominated by *Leptodontidium* sp. C2 BESC 319 g. It is possible that the high abundance of these fungi may cause some suppression of pathogens such as *V. dahliae* but the role of these two fungi in plant protection is still unclear.

## Supporting Information

Figure S1 **Cluster analysis of the 50 most abundant fungal operational taxonomic units (OTUs) in the rhizosphere of four strawberry cultivars, Honeoye, Florence, Senga Sengana and Zephyr, grown in conventionally and organically managed soils or a peat-based growth substrate, with and without** ***Verticillium dahliae*** **.** These OTUs constituted 90% of the total reads in roots and 70% of the reads in rhizosphere soil. The clustering was based on paired group linkage using the Bray-Curtis similarity measure. All OTUs with ≥95% identity are included.
(TIF)

Figure S2 **Non-metric multidimensional scaling (NMDS) analysis of the effects of inoculation with** ***Verticillium dahliae*** **on community structure of fungi colonising the rhizosphere of four different strawberry cultivars, Honeoye, Florence, Senga Sengana and Zephyr, grown in three different soils.**
(TIF)

Figure S3 **Sample based rarefaction curves of all fungal operational taxonomic units (OTUs) in rhizosphere soil and roots of strawberry using Analytic Rarefaction (Ver 1.3, UGA Stratigraphy Lab, USA).**
(TIF)

Figure S4 **Fresh berry weight (g/pot) of four different strawberry cultivars, Honeoye, Florence, Senga Sengana and Zephyr, grown in conventionally and organically managed soils or a peat-based growth substrate, with and without** ***Verticillium dahliae*** **.** Asterisks indicate statistically significant differences (*p*<0.05) between control and *Verticillium dahliae* inoculated treatments within each soil. Vertical bars represent mean values and error bars indicate ±1SE (n = 6).
(TIF)

Table S1 **Similarity percentage (SIMPER) analysis of all fungal operational taxonomic units (OTUs) in rhizosphere and roots of four different strawberry cultivars, Honeoye, Florence, Senga Sengana and Zephyr, grown in conventionally and organically managed soils or a peat-based growth substrate, with and without** ***Verticillium dahliae*** **.** All OTUs with ≥95% identity are included. (ND = not determined).
(DOCX)
