# Supplementary figures and images for: Influence of Soil Type, Cultivar and Verticillium dahliae on the Structure of the Root and Rhizosphere Soil Fungal Microbiome of Strawberry
Source: PLoS One. 2014 Oct 27;9(10):e111455. doi: 10.1371/journal.pone.0111455 (PMC4210224; doi:10.1371/journal.pone.0111455)

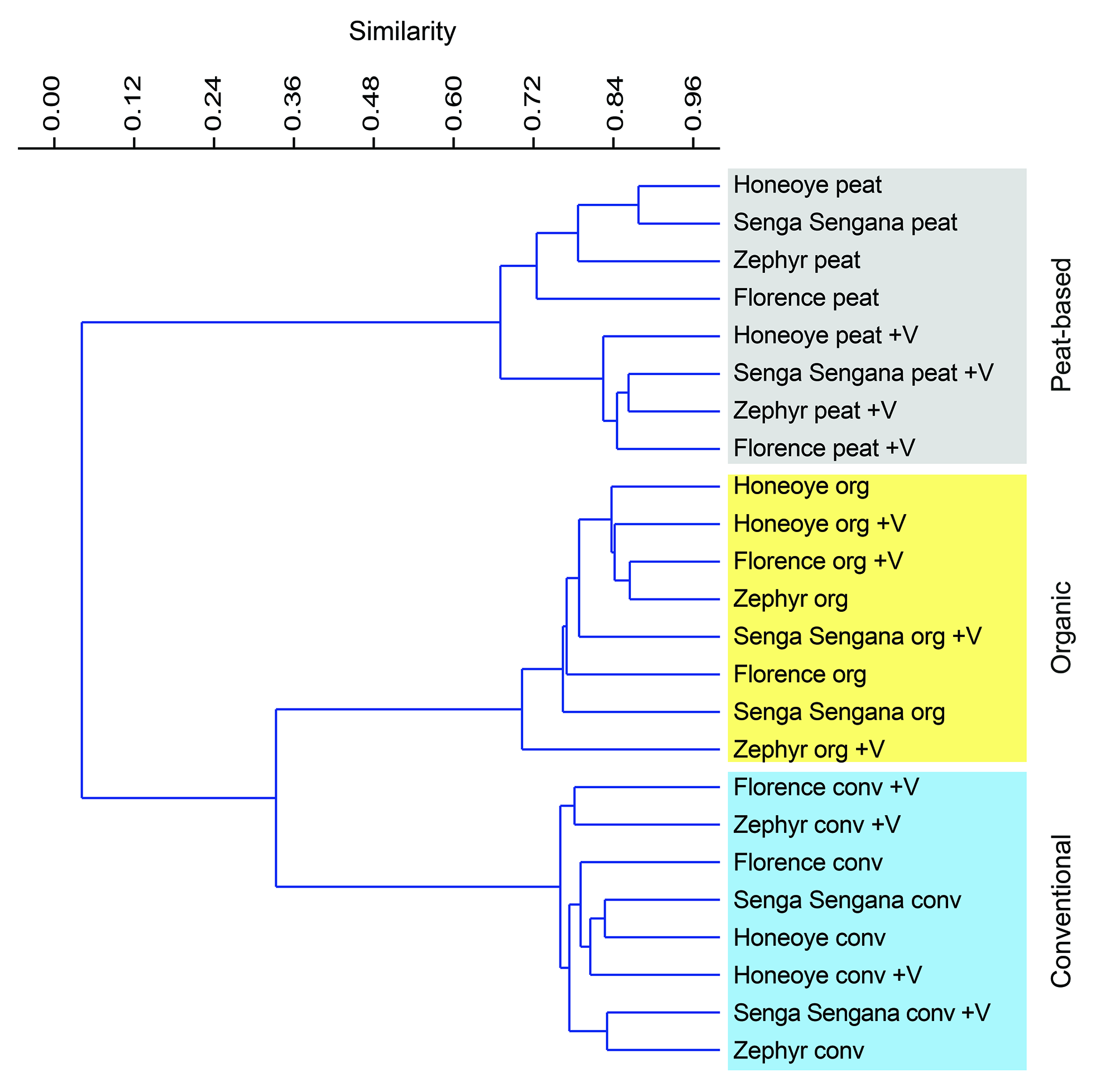

Supplement: Figure S1 — Cluster analysis of the 50 most abundant fungal operational taxonomic units (OTUs) in the rhizosphere of four strawberry cultivars, Honeoye, Florence, Senga Sengana and Zephyr, grown in conventionally and organically managed soils or a peat-based growth substrate, with and without Verticillium dahliae . These OTUs constituted 90% of the total reads in roots and 70% of the reads in rhizosphere soil. The clustering was based on paired group linkage using the Bray-Curtis similarity measure. All OTUs with ≥95% identity are included. (TIF) [file pone.0111455.s001.tif]

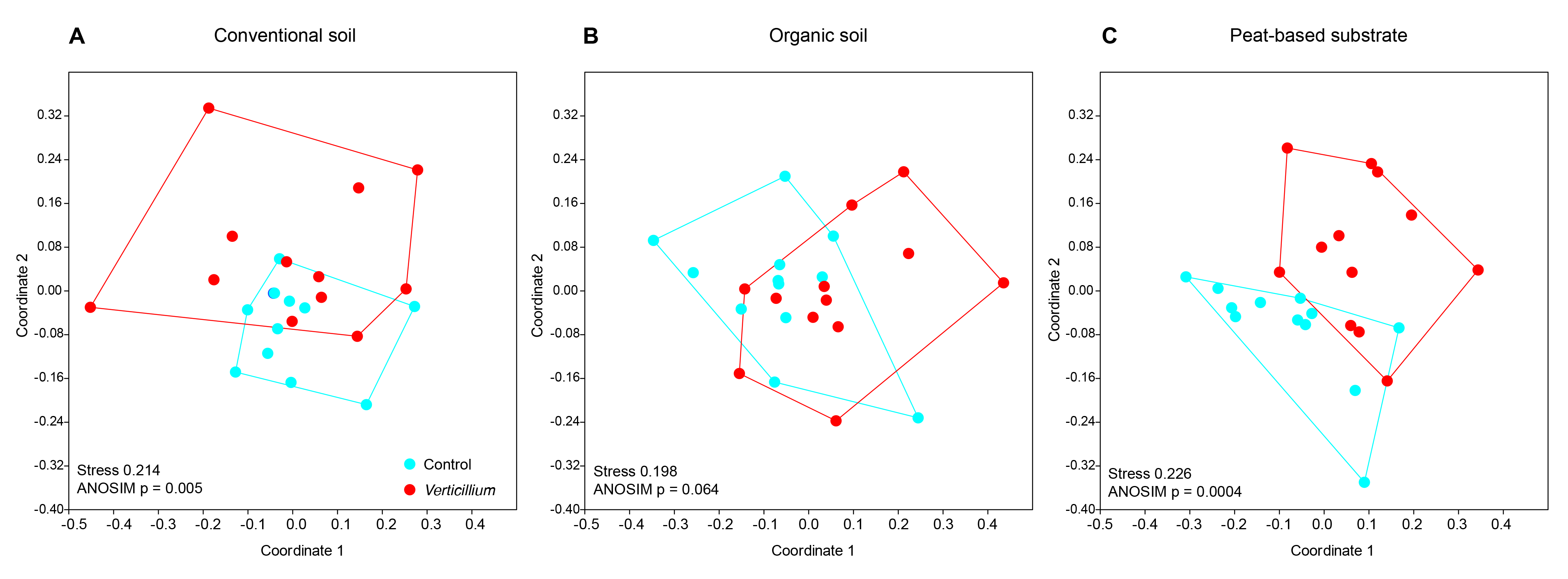

Supplement: Figure S2 — Non-metric multidimensional scaling (NMDS) analysis of the effects of inoculation with Verticillium dahliae on community structure of fungi colonising the rhizosphere of four different strawberry cultivars, Honeoye, Florence, Senga Sengana and Zephyr, grown in three different soils. (TIF) [file pone.0111455.s002.tif]

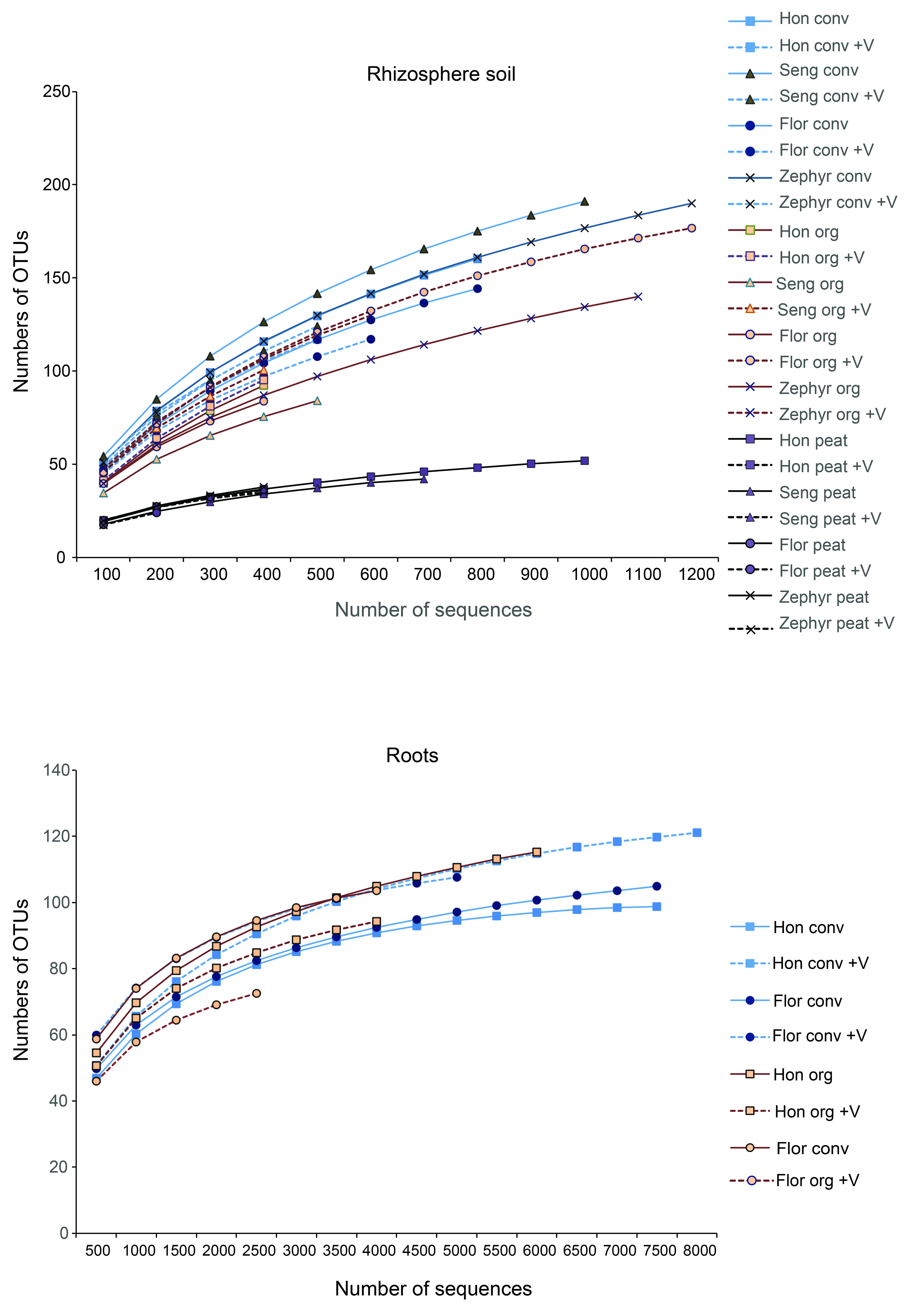

Supplement: Figure S3 — Sample based rarefaction curves of all fungal operational taxonomic units (OTUs) in rhizosphere soil and roots of strawberry using Analytic Rarefaction (Ver 1.3, UGA Stratigraphy Lab, USA). (TIF) [file pone.0111455.s003.tif]

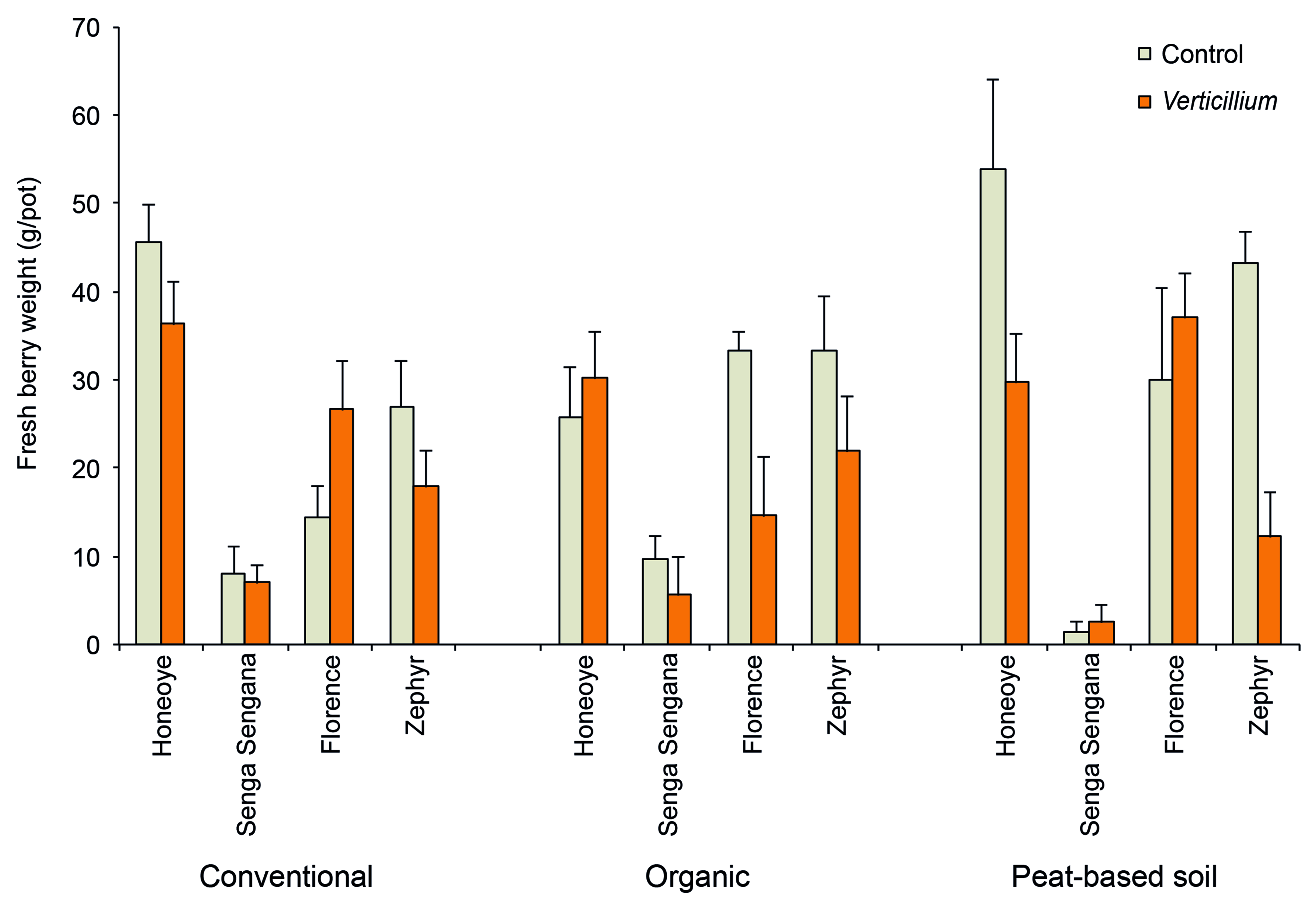

Supplement: Figure S4 — Fresh berry weight (g/pot) of four different strawberry cultivars, Honeoye, Florence, Senga Sengana and Zephyr, grown in conventionally and organically managed soils or a peat-based growth substrate, with and without Verticillium dahliae . Asterisks indicate statistically significant differences (p<0.05) between control and Verticillium dahliae inoculated treatments within each soil. Vertical bars represent mean values and error bars indicate ±1SE (n = 6). (TIF) [file pone.0111455.s004.tif]
