# Supplementary material for: Influence of Soil Type, Cultivar and Verticillium dahliae on the Structure of the Root and Rhizosphere Soil Fungal Microbiome of Strawberry
Source: PLoS One. 2014 Oct 27;9(10):e111455. doi: 10.1371/journal.pone.0111455 (PMC4210224; doi:10.1371/journal.pone.0111455)
Supplement: Table S1 — Similarity percentage (SIMPER) analysis of all fungal operational taxonomic units (OTUs) in rhizosphere and roots of four different strawberry cultivars, Honeoye, Florence, Senga Sengana and Zephyr, grown in conventionally and organically managed soils or a peat-based growth substrate, with and without Verticillium dahliae . All OTUs with ≥95% identity are included. (ND = not determined). (DOCX) [file pone.0111455.s005.docx]

| Environment | Soil | New Cultivars | | Old cultivars | |
| --- | --- | --- | --- | --- | --- |
|  |  | Honeoye  Ctrl x Verti | Florence  Ctrl x Verti | Senga Sengana  Ctrl x Verti | Zephyr  Ctrl x Verti |
| Rhizosphere soil | Conventional | 47.6 % | 52.0 % | 51.6 % | 54.3 % |
|  | Organic | 41.4 % | 46.2 % | 52.0 % | 45.2 % |
|  | Peat-based | 30.9 % | 40.7 % | 44.5 % | 52.0 % |
| Roots | Conventional | 53.9 % | 60.0 % | ND | ND |
|  | Organic | 53.2 % | 70.8 % | ND | ND |
